# Effect of vascularized jejunal conduit flap on peripheral nerve regeneration in rats

**DOI:** 10.55730/1300-0144.5851

**Published:** 2024-05-23

**Authors:** Majid İSMAYİLZADE, Bilsev İNCE, Pembe OLTULU, Zikrullah BAYCAR, Münür Selçuk KENDİR, Mehmet DADACI

**Affiliations:** 1 Department of Plastic, Reconstructive, and Aesthetic Surgery, Faculty of Medicine, İstinye University, İstanbul, Turkiye; 2 Department of Plastic, Reconstructive, and Aesthetic Surgery, Faculty of Medicine, Necmettin Erbakan University, Konya, Turkiye; 3 Department of Pathology, Faculty of Medicine, Necmettin Erbakan University, Konya, Turkiye

**Keywords:** Jejunum conduit flap, peripheral nerve regeneration, vascularized conduit, autologous conduit flap

## Abstract

**Background/aim:**

In the literature, almost all of the nerve conduits proposed for obtaining better nerve recovery were applied as graft materials. In this study, we aimed to propose a new nerve conduit model with a flap pattern and evaluate the effect of a pedicled vascularized jejunal flap on nerve regeneration after wrapping it around a sciatic nerve.

**Materials and methods:**

A total of 90 Wistar albino rats were randomly divided into nine groups with 10 rats in each. The first three groups constituted the control groups, whereas Groups 4–6 were the jejunum conduit (JC)-applied groups. A mucosa-resected JC (MRJC) was applied in Groups 7 and 8. Epineurial neurorrhaphy was performed in Groups 1, 4, and 7; repair with a nerve graft was applied in Groups 2, 5, and 8; and a 1-cm-long nerve defect was created in Groups 3, 6, and 9. After 2 months of follow-up, nerve regeneration was assessed by statistical analyses of the Sciatic Functional Index (SFI) and histopathological evaluation.

**Results:**

The MRJC groups had significantly better results in terms of SFI (p = 0.005). Statistical differences in axonal degeneration, axonal density, myelination, and disorganization were found between all control groups and MRJC groups (p = 0.022, p = 0.001, p = 0.001, and p = 0.039, respectively).

**Conclusion:**

In this study, the feasibility of wrapping around the nerve repair zones of pedicled autologous flaps designed in a tubular fashion was observed in a small rat model. The findings must be further validated with larger animals before clinical testing.

## 1. Introduction

Although numerous studies have investigated peripheral nerve repair, none of the developed techniques have shown superiority over primary end-to-end neurorrhaphy. For injuries involving nerve defects, reconstruction with an autologous nerve graft remains the gold-standard option. However, some factors such as donor area morbidities, scar occurrence, secondary incision requirement, and limited amounts of donor nerve restrict the extensive usage of nerve grafts. In recent years, nerve conduits (nerve guidance channels), which are assumed to be a good alternative to nerve grafts, have been widely sought in nerve regeneration studies supported by the development of biotechnology [1,2].

Only a few studies have investigated the effect of intestinal tissues on the nerve-healing process following the surgical adaptation of these structures as a nerve conduit [3,4]. In the literature, nearly all of the proposed nerve conduits for obtaining better nerve recovery were applied as graft materials without any blood supply. It is well established that vascularization facilitates nerve-healing stages by reducing intraneural fibrosis and increasing the number of Schwann cells and axonal regeneration [5]. In addition, a well-vascularized wound bed inhibits the infiltration of fibroblasts to nerve repair zones and provides an ideal nutritional environment for better healing [6].

In this study, we aimed to propose a new nerve conduit model with a flap pattern and evaluate the effect of a pedicled vascularized jejunal flap on nerve regeneration after wrapping it around a sciatic nerve. After this experimental study conducted in rats, the effects of vascularized pedicled conduit flaps on peripheral nerve regeneration should be validated in larger animal models in terms of clinical applicability. In clinical practice, the use of autologous vascularized conduit flaps may cause additional donor-area morbidities because the most appropriate donor areas for this purpose appear to be the pedicled fascial flaps.

## 2. Materials and methods

This experimental study was conducted between June and August 2020 following the approval of the ethics committee of the Necmettin Erbakan University Meram Faculty of Medicine’s KONÜDAM Experimental Medicine Application and Research Center (Approval No. 2017-034). A total of 90 female Wistar albino rats weighing 250–300 g were used in this study. The rats were randomly divided into nine subgroups, each with 10 rats (Table 1).

**Figure 1:**
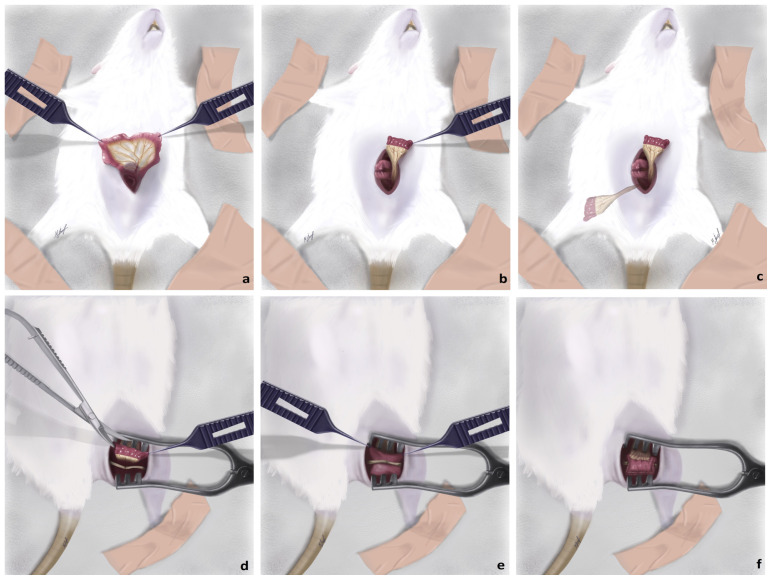
Illustration of the surgical technique performed in this experimental study: **a)** After the abdomen was incised, jejunal loops and the mesojejunum were revealed and separated from the surrounding tissues meticulously. **b)** A 2-cm-long jejunal segment was prepared while keeping the vascular connection intact and jejunal continuity was regained by continuous end-to-end anastomosis of the remaining proximal and distal parts. **c)** The shadowed part indicates the vector of the jejunal flap, which is connecting the intraabdominal space with the sciatic nerve region. **d)** After the jejunal flap was transferred to the sciatic nerve region it was cut longitudinally with microscissors to wrap it around the sciatic nerve (the sciatic nerve was repaired by epineurial neurorrhaphy as in this illustration). **e)** The sciatic nerve was placed into the unwrapped jejunal flap. Mucosal resection was performed at this stage of the experiment for animals in Groups 7, 8, and 9. **f)** The jejunal flap was wrapped around the sciatic nerve and fixed with 8/0 nylon sutures (all illustrations by M. İsmayilzade).

The first three groups (Groups 1, 2, and 3) were the control groups: epineurial neurorrhaphy was performed in Group 1, repair with a nerve graft was done in Group 2, and a 1-cm-long nerve defect was created in Group 3 and bridged with the vein graft. The same surgical procedures were respectively applied for the next three groups (Groups 4, 5, and 6) and a pedicled jejunum conduit (JC) flap was wrapped around the nerve. Epineurial neurorrhaphy + JC flap was applied for Group 4, while a 1-cm nerve graft wrapped with a JC flap was used in Group 5 (Figures 1a–1f). Group 6 consisted of rats with a 1-cm-long nerve defect bridged by the JC flap. The last three groups (Groups 7, 8, and 9) underwent the same respective surgical procedures and a mucosa-resected jejunal conduit (MRJC) flap was wrapped around the nerve. Sciatic nerve transection and epineurial neurorrhaphy were applied in Groups 1, 4, and 7. A 1-cm-long sciatic nerve segment was resected and used as an interpositional nerve graft in all nerve graft groups (Groups 2, 5, and 8). A 1-cm-long defect was created and bridged with the vein graft in Group 3, whereas it was bridged by the JC in Groups 6 and 9.

### 2.1. Anesthesia

All surgical procedures in this study were performed under anesthesia using a combination of xylazine (10 mg/kg Rompun, Bayer, İstanbul, Türkiye) and ketamine (90 mg/kg Ketalar, Eczacıbaşı, İstanbul, Türkiye). Euthanasia of the rats was done by decapitation under anesthesia. To provide analgesia after surgery, 100 mg/kg paracetamol was added to the drinking water of the rats.

### 2.2. Surgical technique

The right sciatic nerves of the rats were used in this study. After a 2-cm incision was made, the sciatic nerve was revealed beneath the biceps femoris muscle. An automatic retractor was used to obtain clear exposure and the sciatic nerve was meticulously dissected from the surrounding tissues under the surgical microscope (f170, OPMI Pico, Carl Zeiss, Jena, Germany).

**Figure 2:**
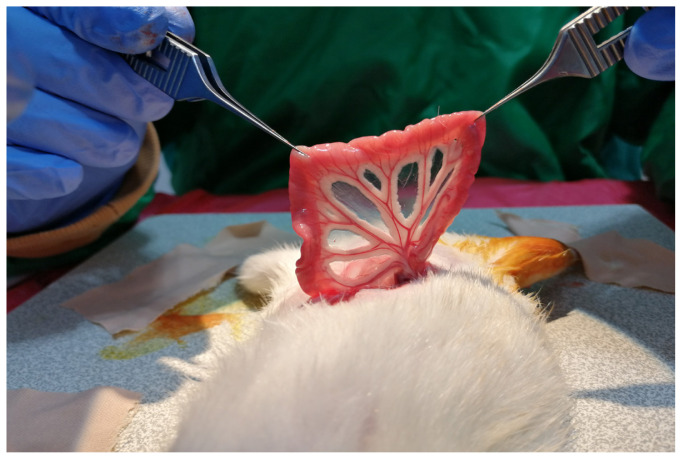
Jejunal loops and mesojejunum are seen.

**Figure 3:**
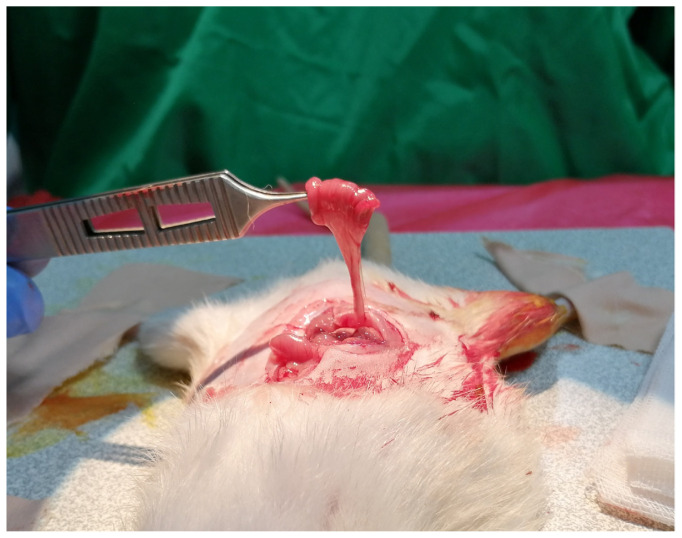
A 2-cm-long jejunal segment was prepared based on the segmental artery.

**Figure 4:**
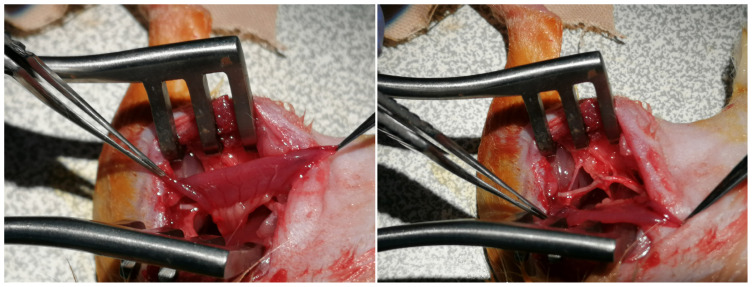
Transposition of the jejunal flap to the sciatic nerve region was achieved through the tunnel created between intraabdominal space and the posterior thigh.

**Figure 5:**
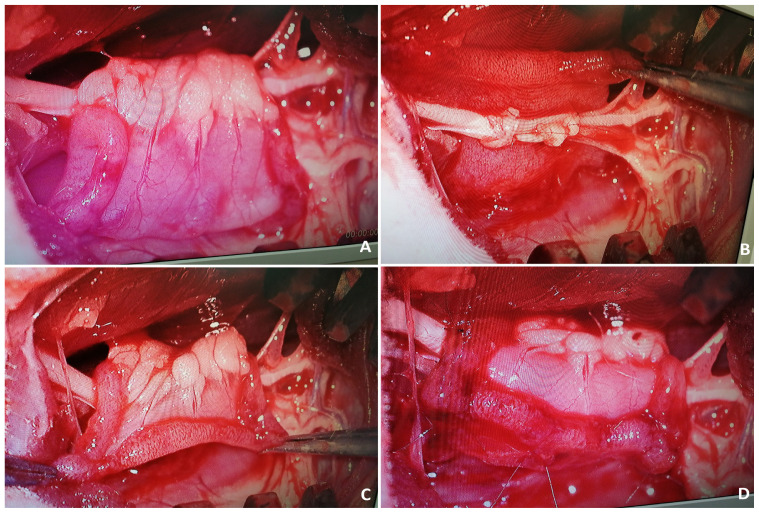
Microscopic views of the jejunal flap and sciatic nerve repaired with a nerve graft. The flap was incised longitudinally and wrapped around the sciatic nerve.

Thereafter, a piece of sterile gauze was placed on the wound and the rat was put into a supine position. A vertical incision of 3–4 cm was made on the abdominal midline skin and subcutaneous tissues were retracted. The intraabdominal space was reached after the midline of the anterior wall muscles of the abdomen and the peritoneum were split. The jejunal loops and mesojejunum were revealed and gently separated from the surrounding tissues (Figure 2). To prevent dehydration of the viscera, 5 mL of saline irrigation was administered at intervals of 10 min during surgical procedures in the intraabdominal region. After designation of the 2 cm of the jejunal segment to be transferred, the supplying artery of the segment was followed up to the jejunal trunk. With only the vascular connection remaining intact, the segment was separated using microscissors both distally and proximally (Figure 3). Jejunal continuity was then regained by continuous end-to-end anastomosis of the proximal and distal jejunal parts with 7/0 propylene suture (Doğsan, Ankara, Türkiye) under a surgical microscope. A tunnel was created between the intraabdominal space and posterior thigh region to transfer the isolated segment to the sciatic nerve area with its vascular pedicle. A mosquito hemostat was passed through the posterior floor of the abdominal wall to the posterior thigh area. Care was taken to form a path of adequate width without pressing on the pedicle. The angle between the sacral bones and the rat’s neutral posture allowed the jejunal segment to be transferred without any tension (Figures 4a and 4b). Following the different surgical procedures for the sciatic nerve in the different groups, the transferred jejunal segment was cut longitudinally and wrapped around the nerve repair zone (Figures 5a–5d).

The mucosa was excised at this stage of the surgery in groups with MRJC flaps. Initially, the mucosa was gripped with microforceps and excised linearly using microscissors. Care was taken not to leave any mucosal part on the whole surface in order to prevent secretion. The longitudinal incision was repaired with 8/0 nylon sutures and the conduit was fixed around the nerve.

The defect size and lengths of the nerve graft and vein graft were 1 cm in all groups; however, the JC was designed to be 2 cm long to enable fixation both distally and proximally. A 1-cm nerve segment was excised and readapted in the inverted direction as a nerve graft, whereas a 1-cm vein graft was obtained from the right external jugular vein. A 8/0 Dylon suture was then used in all surgical procedures performed for the sciatic nerve.

### 2.3. Assessment of nerve regeneration

Nerve regeneration was evaluated 2 months (60 days) after surgery by walking track analysis and histopathological assessment.

#### 2.3.1. Walking track analysis

After 2 months of follow-up, all rats were made to walk on a walking platform of 100 × 40 × 20 cm. The Sciatic Functional Index (SFI) was calculated according to the formula described by de Medinaceli et al. and modified by Bain et al. [7].

#### 2.3.2. Histopathological evaluation

Samples of 1 cm in size, including the nerve repair zones and the conduits around them, were taken for the histopathological assessment of nerve regeneration. All samples were fixed in 10% paraformaldehyde and embedded in paraffin blocks. They were cut into 5-μm cross-sections and transverse sections using a microtome (RM2125, Leica, Wetzlar, Germany), and four pieces were placed on lysine-coated slides, which were stained with hematoxylin and eosin, CD34, Luxol fast blue, and Masson’s trichrome. A total of 12 sections with three sections for each histological marker were analyzed. To visualize the axonal fibers, tissue sections were incubated with the antibodies of purified antineurofilament markers (panaxonal and cocktail) at a dilution of 1:800. S100 protein was used at a dilution of 1:200 to stain the myelin sheets. An optical microscope (BX51, Olympus, Tokyo, Japan) was used to evaluate the following parameters for measuring nerve regeneration: edema, fibrosis, inflammation, vascularization, axonal degeneration, axonal density, myelination, and disorganization. Two pathologists blinded to the groups evaluated the materials and gave a score between 0 and 3. Vascular structures and nerve fibers were manually counted at 200× magnification. Because the parameters of edema, axonal degeneration, disorganization, myelination, fibrosis, and inflammation are uncountable, these parameters were measured according to a semiquantitative scoring system as follows: 1 point, mild; 2 points, moderate; and 3 points, severe [8].

### 2.4. Statistical analysis

Statistical analysis was performed using IBM SPSS Statistics 24.0 for Windows (IBM Corp., Armonk, NY, USA). Data were evaluated for compliance with normal distribution. The sample size was determined with a standardized difference of 0.5 and a power level (pβ) of 0.8 (accepted level of type II error). Analysis of variance was used to analyze normally distributed variables. Kruskal–Wallis analysis of variance was performed for nonnormally distributed variables. A Bonferroni-corrected Mann–Whitney U test was used for binary comparisons and p < 0.05 was considered as the significance level. Initially, comparison studies were performed between the three main groups according to the surgical techniques. Further statistical analyses were then conducted between the subgroups.

## 3. Results

After 2 months of follow-up, the SFI was calculated following walking track analysis. Pressure sores were not observed in any of the rats. Complications were not encountered in any surgical sites. Because of the intraabdominal intervention, the mean weight of the rats was 251.4 g (range: 200.3–309.9 g) at the end of 2 months. Seven rats that died in the early postoperative period were replaced with new animals. In five rats (two rats each in Groups 4 and 5; one rat in Group 6), macroscopic cyst formation consisting of homogenic dense liquid was observed during the surgical exploration of the sciatic nerves and conduits. No herniation of the intraabdominal structures was observed and there was no case of autotomy of the injured nerve-related extremity in our study. SFI values and histopathological assessment results are demonstrated in Table 2.

**Figure 6:**
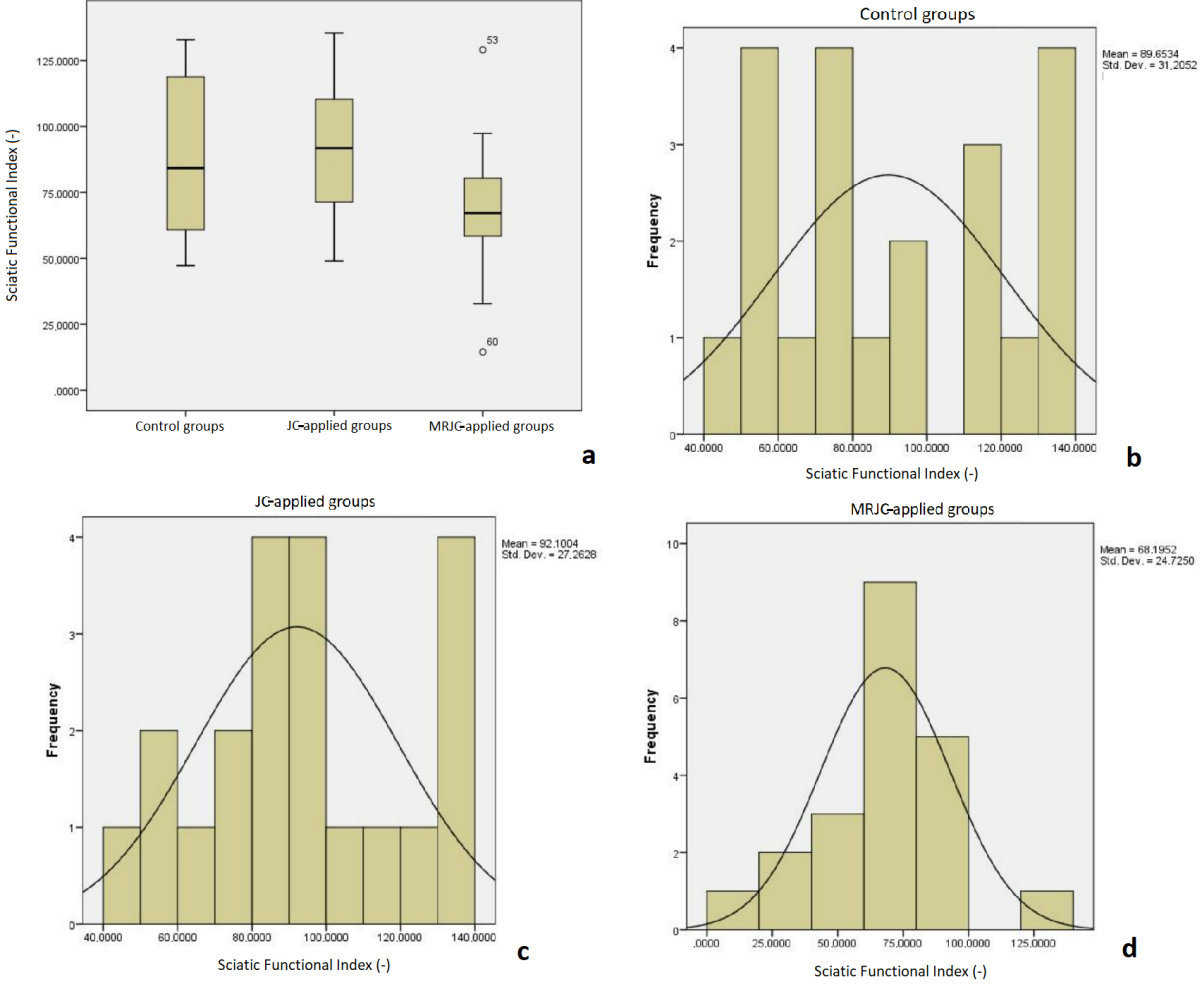
SFI results of the three main treatment groups: **a)** Significant difference was revealed in the rats with MRJCs; frequency distributions for **b)** control groups, **c)** JC-applied groups, and **d)** MRJC-applied groups (SFI: Sciatic Functional Index; JC: jejunum conduit; MRJC: mucosa-resected jejunum conduit).

The mean SFI values were as follows: Group 1, 101.4 ± 16.4; Group 2, 92.8 ± 11.8; Group 3, 74.8 ± 10.1; Group 4, 90.9 ± 13.5; Group 5, 91.6 ± 17.3; Group 6, 93.8 ± 12.6; Group 7, 78.2 ± 9.7; Group 8, 54.9 ± 6.9; and Group 9, 71.4 ± 8.3. Statistical analyses were first performed between the three main treatment groups (control, JC flap, and MRJC flap groups) (Figure 6a).

The groups undergoing JC surgery with mucosa resection had significantly better SFI values (p = 0.022) (Figures 6b–6d). In studies involving paired comparisons (Mann–Whitney U test), a significant difference was seen between all of the JC groups and the MRJC groups (p = 0.005). The SFI results of the MRJC groups were also better than those of the control groups; however, the differences between the vein graft group (Group 3) and the MRJC groups were not significant (p = 0.277).

**Figure 7:**
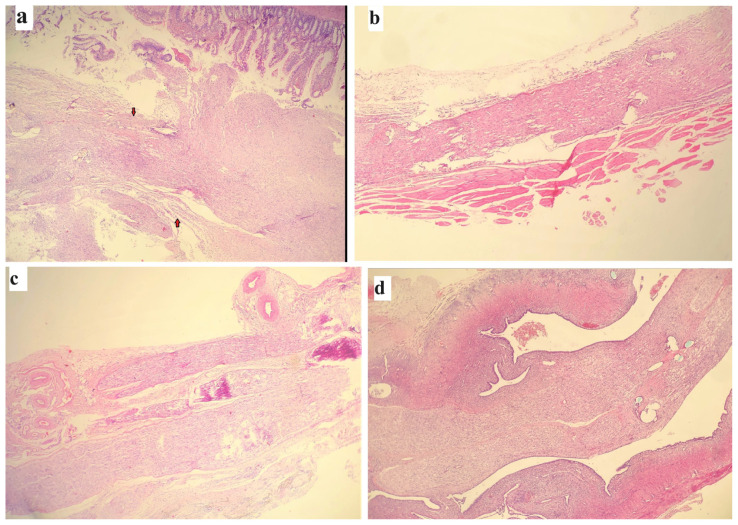
Histopathological views of hematoxylin and eosin staining: **a)** In the group with epineurial neurorrhaphy + jejunum conduit flap (Group 4), the intestinal mucosa is shown by the upper red arrow, while the lower red arrow indicates irregular nerve fibers. **b)** Neuroma occurrence is shown in Group 2 (repair via nerve graft). **c)** Regular nerve fibers are seen in the vein graft group (Group 3). **d)** Minimal inflammation and regular transition of the nerve fibers are demonstrated in the group with nerve graft + mucosa-resected jejunum conduit (Group 8).

In all groups, lymphocytes were the dominant inflammatory cells, but clustered mixtures of neutrophils, lymphocytes, and plasmacytes were observed in Groups 4 and 6. All groups that underwent JC surgery had worse histopathological results compared to both the control groups and the MRJC groups (Figures 7a–7d).

**Figure 8:**
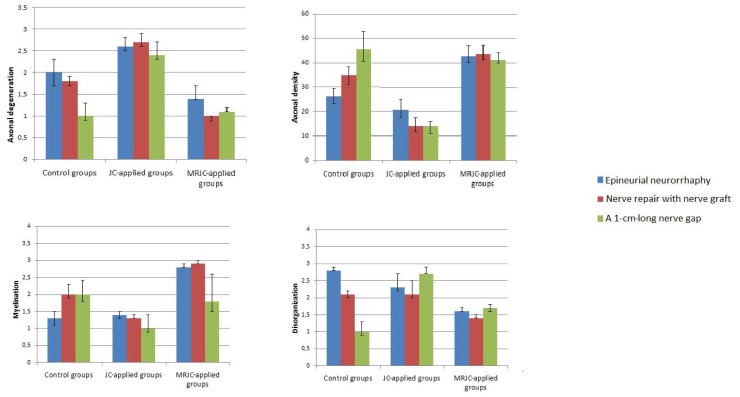
Significant differences were found for the histopathological parameters of axonal degeneration, axonal density, myelination, and disorganization. Axonal density was evaluated by the number of axonal fibers per unit, whereas axonal degeneration, myelination, and disorganization were scored between 0 and 3 by pathologists (JC: jejunum conduit; MRJC: mucosa-resected jejunum conduit).

Statistical differences between Groups 1 and 2 (control groups) and Groups 7 and 8 (MRJC groups) were significant in terms of the parameters of axonal degeneration, axonal density, myelination, and disorganization (Figure 8).

The axonal degeneration values were 2 ± 0.8 in the epineurial neurorrhaphy group (Group 1) and 1.8 ± 0.2 in the nerve graft group (Group 2), whereas they were 1.4 ± 0.2 in the epineurial neurorrhaphy + MRJC group (Group 7) and 1 ± 0.1 in the nerve graft + MRJC group (Group 8). Significant differences were found between Groups 1 and 7 (p = 0.031) and between Groups 2 and 8 (p = 0.022). The axonal density values were 26.1 ± 6.3 in Group 1, 35 ± 7.5 in Group 2, 42.6 ± 6.9 in Group 7, and 43.7 ± 5.8 in Group 8. A significance value of p = 0.001 was obtained for the comparisons between Groups 1 and 7 and between Groups 2 and 8. The myelination values were 1.3 ± 0.4 and 2 ± 0.4 in Groups 1 and 2, respectively, whereas they were 2.8 ± 0.1 in Group 7 and 2.9 ± 0.1 in Group 8. Statistical analysis revealed significance of p = 0.001 for both comparisons. The comparisons between disorganization values in Groups 1 and 7 and those in Groups 2 and 8 revealed significance of p = 0.039 and p = 0.044, respectively.

## 4. Discussion

In this study, the autogenous jejunal tissues of rats were transferred to the nerve repair zone with the original vascular connection in contrast to other biological conduits that were previously described as grafts. The jejunal tissue, which has a segmental pattern of blood supply, was prepared based on the most appropriate jejunal branch. Although the use of jejunal and intestinal tissues does not yet appear to have clinical applicability, we aimed to demonstrate the effects of a “living” conduit on peripheral nerve regeneration in a vascularized nerve conduit model. Several studies have reported the favorable effects of nonvascularized omentum grafts in cases of peripheral nerve injuries and omentum flaps in cases of spinal cord injuries [9,10]. The main goal of our study was to determine the significance of a vascularized tubular flap in peripheral nerve regeneration because the anatomical distance and fascial structure of the omentum was not preferred. The longitudinal muscles of the rat jejunum allowed these tubular structures to protect the tonus even when they were cut longitudinally; thus, JC collapse was not encountered. In six of the nine groups, jejunal tissues were transferred to the sciatic nerve area as a nerve conduit and the intraabdominal connection remained intact. In the literature, a previous study investigated the effects of a vascularized fallopian tube flap on nerve regeneration and demonstrated that flap usage could result in failure. It was associated with the continuity of physiological secretion in the flap conduits causing cysts and intense inflammatory contents [11]. Therefore, to block excretion, we resected the inner mucosa of the jejunum following the longitudinal incision in the MRJC groups, while in the JC groups, the mucosal structure was saved to demonstrate the effects of multiple villi on peripheral nerve regeneration. However, although the ciliary epithelium was expected to be effective, it failed because of secretions.

In the literature, nerve guidance channels are generally applied as graft materials [12–14]. Additionally, in vivo and in vitro studies have been performed to enrich biological and synthetic conduits, adding several trophic factors, and to obtain more promising results in nerve regeneration. It is well known that the blood supply in a wound bed is one of the most important factors for successful regeneration. Moreover, studies have examined the effects of vascularization and angiogenesis on nerve graft healing [15,16]. Studies have also reported that superficial inferior epigastric artery fascia flaps wrapped around decellularized nerve allografts facilitated functional recovery by increasing angiogenesis [17,18]. The need for a nerve graft frequently arose in cases with crush or delayed injuries in clinical practice. These nerve injuries are accompanied by severe inflammation, deficient blood supply, and multiple foreign bodies. Accordingly, injuries including nerve gaps require a well-supplied wound bed to support the viability of the nerve graft. The main objective of our study was to present the favorable effects of a well-supplied tubular structure on peripheral nerve regeneration in epineurial neurorrhaphy, repairs obtained with nerve grafts, and injuries with a nerve gap. In this way, conduits with a pedicled pattern would neither need extra blood supply in a transferred area nor be influenced by unfavorable conditions of the wound bed. Yi et al. developed a nerve guidance channel using acellular porcine small intestinal mucosa and investigated its effect on sciatic nerve regeneration in rats [4]. The small intestinal mucosa was reported to facilitate nerve regeneration with its nerve growth factor-like effects and was suggested as a possible good alternative to autologous nerve grafts in further clinical studies. In another study, a 15-mm ileal segment was obtained via intraabdominal intervention and compared with an autologous nerve graft in terms of nerve regeneration effects. The benefits of the ileal segment placed as a graft conduit were observed; however, a statistical comparison revealed that the autologous nerve graft achieved significantly better results [3].

With biomedical engineering developments, several companies have conducted investigations of 3D-designed biodegradable scaffolds to find alternatives to autografting. Angiogenesis and inosculation are two principal vascularization strategies in tissue engineering. Inosculation is described as a microvascular network preformed in scaffolds with several methods, such as the seeding of endothelial progenitor cells, whereas angiogenesis is the ingrowth process of vascular structures into an implanted scaffold or repair zone [19]. For angiogenesis, vascular endothelial growth factors (VEGFs) [20,21], basic fibroblast growth factors [22,23], platelet-derived growth factors [24], and angiogenin [25] are the most frequently used factors. Additionally, VEGFs are used in peripheral nerve regeneration studies and the activation of angiogenesis; however, studies on the inosculation strategy are very scarce. Although they are not yet ready for use in clinical applications, 3D-designed and manufactured capillary-like networks in scaffolds are likely to obtain promising results to solve blood supply problems in the future [19,26].

Significant discrepancies were found in the comparisons of the histopathological results in terms of axonal degeneration, axonal density, myelination, and disorganization. No difference was noted between the MRJC and control groups regarding edema, fibrosis, inflammation, and vascularization. These findings support alterations in axonal regeneration rather than common tissue-healing processes. Considering the progression of the only parameters that were directly associated with nerve healing, the findings can be attributed to the fact that an adequate blood supply makes Schwann cells proliferate and migrate to the nerve repair zone [5]. However, there were inconsistencies in vascularization between the groups. The pedicled jejunal flap was wrapped around the sciatic nerve as a conduit in six of the nine groups. Although increased vascularity was determined in all groups, it appears that vascularization alone is not a strong determinant for healthy peripheral nerve regeneration. The nerve-healing process is compatible with wound-healing processes, and inflammation should not be underestimated in this context. Intensive inflammation due to secretions in Groups 4–6 affected many blood vessels in the healing process. However, this vascularization did not contribute to peripheral nerve healing. Microcomputed tomography studies have shown that the main revascularization during the nerve-healing period occurs as endoneurial longitudinal inosculation from the proximal to the distal segment [27,28]. When the metabolic requirements of nerve healing exceed the longitudinal inosculation ability, centripetal revascularization occurs from the surrounding bed [29]. Vascularization increases Schwann cell migration, facilitates axonal growth, and decreases intraneural fibrosis [5]. However, vascularization alone remains insufficient for nerve regeneration. Lee et al. stated that although VEGF triggered revascularization, it did not cause a significant difference in functional motor recovery and nerve-conduction velocity [30]. Some authors assume that a supportive microenvironment after nerve injury, including a stable blood supply, is much more important than VEGF-induced revascularization. In our study, the vascularization level in Group 1 was high, whereas the SFI values were unsatisfactory. Although it has not been mentioned in pathology studies before, vascularization accompanied by a high level of inflammation is unlikely to contribute to peripheral nerve regeneration.

In the present study, the vein graft group (Group 3) was created to obtain a standardized comparison with the groups in which JCs and MRJCs were wrapped around the nerve repair zone with a 1-cm gap (Groups 6 and 9). The use of vein grafts for bridging nerve gaps has been addressed thoroughly in the literature [31]. In this study, we used the right external jugular vein in Group 3 to bridge the defects harvested from the right cervical area following a 2-cm-long vertical-oblique incision. The main obstacle of a vein graft is its inevitable collapse when it is applied to bridge defects longer than 2.5–3 cm. No significant difference was found between the vein graft and MRJC groups in terms of functional recovery and histopathological parameters. In addition, the nerve-healing parameters of the rats with vein grafts were significantly better than those of rats with primary epineurial neurorrhaphy and autologous nerve grafts. In the literature, small gaps deliberately created in the repair zone have promoting effects on nerve regeneration processes within the framework of nerve selection theory, or neurotropism, with superiority over epineurial neurorrhaphy in experimental rat models [32,33]. Because the increase in the length of the gap will restrict the use of vein grafts, our conduit model can be a surgical alternative in appropriate cases.

A limitation of this study is the relatively short timeframe with respect to other sciatic nerve injury models. The majority of experimental nerve studies terminate the observation period after 12 weeks, whereas others take this period as 8 weeks. There is no consensus about the timeframe; it is generally determined according to the methodology of the study. Considering the dimension of surgical stress in our study, we decided to restrict the timeframe to 8 weeks. This study was designed to demonstrate the benefits of pedicled JCs; thus, creating a poorly vascularized wound bed would have been better to reveal obvious statistical differences. However, all wounds were created in virgin territory and so improvements after the addition of vascularized tissues around the nerve repair site or nerve graft might have been unclear. Further studies investigating the use of pedicled vascularized conduit flaps in irradiated wound beds or surgical areas with poor vascularity can potentially build on these outcomes.

According to the findings of our experimental study, designing human fascia flaps (e.g., anterolateral thigh fascia flaps, serratus fascia flaps, temporoparietal fascia flaps, and radial forearm fascia flaps) in a pedicled or free pattern and wrapping them around the nerve repair zone may provide promising outcomes in clinical practice. Furthermore, pedicled or vascularized conduit flaps will minimize the occurrence of necrosis, which is highly possible with graft conduits applied to inappropriate wound beds. Additionally, free or pedicled vascularized conduit flaps can contribute to the nerve-healing process of mutilating injuries such as brachial plexus injuries.

## 5. Conclusion

Both functional and histopathological nerve recoveries were obvious in the MRJC groups. The use of pedicled and vascularized conduit flaps demonstrated significant results; however, intestinal tissue conduits placed as grafts had no substantive effects on nerve regeneration. On the contrary, vascularization alone was found inadequate in terms of peripheral nerve regeneration in the JC groups. Thus, the determinative effect of inflammation should also be considered. This study has presented the applicability of autogenous tissues as vascularized conduit flaps, which are malleable for being shaped in a tubular pattern and have facilitating effects on peripheral nerve regeneration. Finally, the feasibility of wrapping pedicled autologous flaps designed in a tubular pattern around nerve repair zones has been observed in a small rat model in this study, but it must be further validated in larger animals before clinical testing.

## Figures and Tables

**Table 1:** Distribution of groups with 10 rats in each.

Main treatments	Groups	Rats (n)	Surgical procedure
Control groups	Group 1	10	Epineural neurorrhaphy
	Group 2	10	Epineural neurorrhaphy with nerve graft
	Group 3	10	Vein graft usage to bridge a 1-cm nerve gap
Jejunum conduit (JC)-applied groups	Group 4	10	Epineural neurorrhaphy + JC
	Group 5	10	Epineural neurorrhaphy with nerve graft + JC
	Group 6	10	JC usage to bridge a 1-cm nerve gap
Mucosa-resected jejunum conduit (MRJC)-applied groups	Group 7	10	Epineural neurorrhaphy + MRJC
	Group 8	10	Epineural neurorrhaphy with nerve graft + MRJC
	Group 9	10	MRJC usage to bridge a 1-cm nerve gap

**Table 2:** Mean ± SD values of SFI and histopathological parameters in the groups. The SFI results of the MRJC subgroups were better than those of the control groups; however, the differences between the vein graft group (Group 3) and MRJC groups were not significant (p = 0.277). All subgroups that underwent JC surgery had worse histopathological results than the control groups or the MRJC groups. Statistical differences between Groups 1 and 2 (control groups) and Groups 7 and 8 (MRJC groups) were significant for the parameters of axonal degeneration, axonal density, myelination, and disorganization (SD: standard deviation; SFI: Sciatic Functional Index; JC: jejunum conduit; MRJC: mucosa-resected jejunum conduit).

Groups	SFI	Edema	Inflammation	Fibrosis	Vascularization	Axonal degeneration	Axonal density	Myelination	Disorganization
**Control groups**									
**1**	−101.4 ± 16.4	1 ± 0.2	1 ± 0.5	1.4 ± 0.3	30.1 ± 9.9	2 ± 0.8	26.1 ± 6.3	1.3 ± 0.4	2.8 ± 0.1
**2**	−92.8 ± 11.8	0	1 ± 0.2	1 ± 0.6	15 ± 7.2	1.8 ± 0.2	35 ± 7.5	2 ± 0.4	2.1 ± 0.2
**3**	−74.8 ± 10.1	0	1.1 ± 0.6	1 ± 0.4	15.6 ± 4.2	1 ± 0.4	45.4 ± 12.4	2 ± 0.6	1 ± 0.4
**Jejunum conduit groups**									
**4**	−90.9 ± 13.5	2 ± 0.9	2.6 ± 0.3	2.1 ± 0.4	19.6 ± 9.1	2.6 ± 0.3	20.8 ± 7.7	1.4 ± 0.2	2.3 ± 0.5
**5**	−91.6 ± 17.3	2 ± 0.7	2.7 ± 0.2	2.6 ± 0.1	19.3 ± 6.2	2.7 ± 0.3	14 ± 5.8	1.3 ± 0.1	2.1 ± 0.5
**6**	−93.8 ± 12.6	2 ± 0.7	2.4 ± 0.2	2.7 ± 0.2	33.8 ± 10.8	2.4 ± 0.4	14.1 ± 5.3	1 ± 0.5	2.7 ± 0.2
**Mucosa**-**resected jejunum conduit groups**									
**7**	−78.2 ± 9.7	0	1.3 ± 0.4	1.4 ± 0.3	17.4 ± 8.9	1.4 ± 0.2	42.6 ± 6.9	2.8 ± 0.1	1.6 ± 0.1
**8**	−54.9 ± 6.9	0.7 ± 0.1	1.3 ± 0.5	1 ± 0.3	18.3 ± 7.4	1 ± 0.1	43.7 ± 5.8	2.9 ± 0.1	1.4 ± 0.1
**9**	−71.4 ± 8.3	1 ± 0.1	1.4 ± 0.2	1.4 ± 0.6	18.3 ± 8.3	1.1 ± 0.1	41.1 ± 4.1	1.8 ± 1.1	1.7 ± 0.2
